# Angiogenesis in metastatic colorectal cancer and the benefits of targeted therapy

**DOI:** 10.1186/1756-8722-5-63

**Published:** 2012-10-11

**Authors:** Weijing Sun

**Affiliations:** 1 University of Pittsburgh School of Medicine, UPMC Cancer Pavilion, 5150 Centre Avenue, Fifth Floor, Pittsburgh, PA, 15232, USA

**Keywords:** Angiogenesis, Vascular endothelial growth factor, Colorectal cancer, Antiangiogenic

## Abstract

The diverse pathways and molecules involved in angiogenesis, the formation of new blood vessels, have been targeted for the treatment of colorectal and other cancers. Vascular endothelial growth factor (VEGF)-A binding to VEGF receptor (VEGFR)-2 is believed to be the key signaling pathway mediating angiogenesis. Other VEGF pathways involved in angiogenesis include VEGF-A, VEGF-B, and placental growth factor binding to VEGFR-1, and VEGF-C and VEGF-D binding to VEGFR-2 and VEGFR-3. VEGF signaling also intersects with other pathways, including angiopoietin/Tie, Notch, hypoxia-inducible factor, and integrin pathways. The roles of these pathways in tumor angiogenesis and in various human cancers will be explored in this article. In addition, preclinical and clinical data on bevacizumab, aflibercept (known as ziv-aflibercept in the US), and investigational antiangiogenic agents in development for the treatment of colorectal and other cancers will be reviewed.

## Introduction

Angiogenesis refers to the formation of new blood vessels [1]. As blood vessels are needed to supply nutrients and oxygen to tissues [2], angiogenesis plays an essential role in normal growth and development as well as in the development of cancer [1,3,4]. In normal development, angiogenesis is needed for embryonic development, bone formation, and the function of ovaries and other endocrine glands [4]. In cancer, angiogenesis is required for tumor growth and metastasis [1,3].

Angiogenesis is a highly regulated, complex process orchestrated by a number of intersecting pathways, including vascular endothelial growth factor (VEGF), angiopoietins, Notch, and integrins. In normal tissues, there is a balance between proangiogenic and antiangiogenic factors [2]. During physiologic angiogenesis in adults, there is a temporary shift to proangiogenic factors; this is held in check by inhibitory mechanisms [1,2]. In contrast to normal angiogenesis, tumor angiogenesis is outside of the control of normal physiologic inhibition, and there is an imbalance of proangiogenic and antiangiogenic factors [1,2]. The diversity of pathways and molecules involved in angiogenesis may offer avenues for therapeutic intervention. This article aims to provide an overview of tumor angiogenesis, focusing on the role of VEGF. It will also review preclinical and clinical evidence for the use of antiangiogenic agents in the treatment of colorectal and other cancers.

### Tumor angiogenesis and the role of VEGF

Tumor angiogenesis typically involves the formation of new blood vessels from pre-existing vessels in a process known as sprouting angiogenesis [1,5]. Tumors may also co-opt pre-existing vessels [6]. Compared with the normal vasculature, the tumor vasculature is abnormal in a number of different ways. There is loss of the normal vascular hierarchy of arterioles, capillaries, and venules [7,8]. The endothelial layer is irregular and contains spaces that contribute to the leakiness of tumor vessels [9], proliferating tumor cells cause compression of vessels [10], and interstitial pressure is increased [11]. The ability of tumor vessels to supply oxygen and remove waste products is thus compromised, resulting in hypoxia and acidosis in the tumor microenvironment [12,13]. Abnormalities are also observed in other tumor vasculature components such as pericytes and the basement membrane. Pericytes have an abnormally weak attachment with endothelial cells and have cytoplasmic processes that extend away from the vessel wall [8]. The basement membrane is variable in thickness, has a loose association with endothelial cells and pericytes, and has extensions away from the vessel wall [14]. Endothelial cells in tumor vessels also show altered gene expression [15,16] and cytogenetic abnormalities such as aneuploidy [17].

#### The VEGF signaling pathway

**Figure 1:**
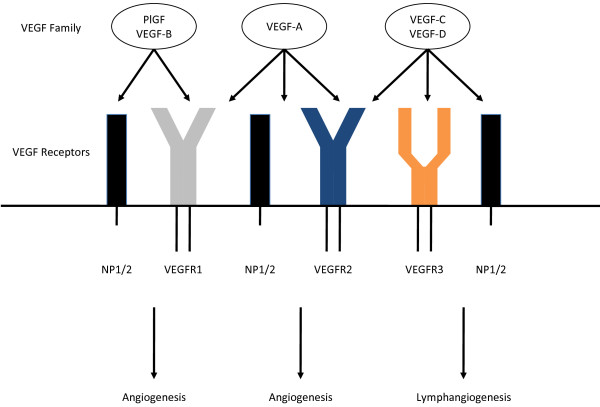
**The VEGF family.** Positive and negative modulation of angiogenesis by VEGFR1 ligands [26].

The mammalian VEGF signaling pathway includes 5 glycoproteins from the VEGF family (VEGF-A, VEGF-B, VEGF-C, VEGF-D, and placental growth factor [PlGF]), 3 receptors (VEGF receptor [VEGFR]-1 [FLT-1], VEGFR-2 [FLK-1/KDR], and VEGFR-3 [FLT-4]), and 2 co-receptors (neuropilin [NRP]-1 and NRP-2) (Figure 1) [5,18-26]. In addition, there are multiple isoforms of VEGF-A (VEGF_121_, VEGF_165_, VEGF_189_, and VEGF_206_) [5]. The receptors for the various VEGF ligands are tyrosine kinases and are found primarily on vascular endothelial cells [18,26].

**Figure 2:**
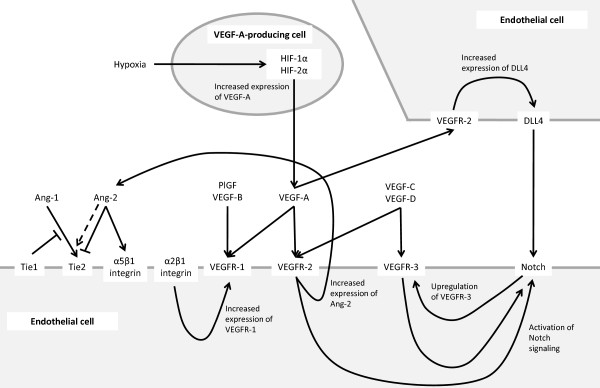
**Selected crosstalk between VEGF and other signaling pathways.** VEGF signaling intersects with a number of other signaling pathways, including Ang/Tie, Notch/Dll4, integrins, and hypoxia to orchestrate tumor angiogenesis [26,27,31-35]. Ang = angiopoietin; Dll4 = delta-like ligand 4; HIF = hypoxia-inducible factor; PlGF = placenta growth factor; VEGF = vascular endothelial growth factor; VEGFR = vascular endothelial growth factor receptor. Dashed arrow indicates Ang-2 can serve as a partial Tie-2 agonist under certain conditions.

VEGF-A binding to VEGFR-2 is believed to be the key signaling pathway mediating angiogenesis [18,27]. VEGF-A enhances endothelial cell proliferation and survival, promotes endothelial cell migration, increases vascular permeability, and alters gene expression [5,18]. Levels of VEGF-A have been shown to be increased in many cancers, including colorectal, prostate, and breast [28]. Downstream signaling activated by VEGF-A binding to VEGFR-2 includes the PLC-gamma/PKC/Ras/Raf/MEK/MAPK signaling pathway [29,30]. Other VEGF family members, along with other signaling mediators, affect and overlap with the function of VEGF-A [26,31,32]. For instance, PlGF, fibroblast growth factor (FGF), and platelet-derived growth factor (PDGF) can modulate the effects of VEGF-A on angiogenesis, and VEGF-A can form heterodimers with PlGF or VEGF-B [26]. Because of alternative splice variants, an enormous diversity of heterodimerization with different functional properties may be formed, which presents a challenge to assessing the functional consequences. In addition, VEGF-A has overlapping function with VEGF-C and VEGF-D [26]. VEGF-A-mediated signaling also intersects with angiopoietin (Ang)/Tie, Notch/delta-like ligand (Dll)4, hypoxia-inducible factor (HIF)-1α and HIF-2α, and integrin pathways [26,31,32] (Figure 2).

Like VEGFR-2, VEGFR-1 is expressed on vascular endothelial cells, but it is also expressed on other cell types, including monocytes and macrophages [26,36]. The affinity of VEGFR-1 for VEGF-A is greater than that of VEGFR-2, although it has weak kinase activity, and it is thought to be a negative regulator of angiogenesis [37]. In cancer, VEGFR-1 appears to play a role in the epithelial-to-mesenchymal transition [38,39]. VEGF-B binding to VEGFR-1 was shown to be associated with increased microvascular density in oral squamous cell carcinoma [40], but it was not associated with tumor vascularity in breast cancer [41] and it inhibited tumor growth in a mouse pancreatic neuroendocrine tumor model [42]. In a non-tumor model of pathological angiogenesis, VEGF-B promoted survival of endothelial cells, pericytes, and smooth muscle cells and upregulated the expression of prosurvival genes [43]. VEGF-B shows increased expression in multiple cancers, including ovarian, colorectal, renal cell, and prostate; its expression is associated with disease stage and expression of its receptor, VEGFR-1, predicts poor prognosis [36].

PlGF, another ligand for VEGFR-1, is thought to play a role in the angiogenic switch in pathological conditions [44]. PlGF binding to VEGFR-1 increases VEGF-A expression [45] and has a synergistic effect on VEGF-A signaling in cancer and other pathological forms of angiogenesis [44]. PlGF appears to promote the growth of tumor cells in an autocrine/paracrine manner [46]. PlGF has been shown to be upregulated in and prognostic for cancers, including gastric, colorectal, lung, breast, renal cell, hepatocellular, and brain [36].

VEGF-C and VEGF-D bind to the receptors VEGFR-2 and VEGFR-3 [26]. VEGF-C expression is associated with advanced metastatic disease for colorectal cancer [47] and it plays a role in lymphangiogenesis and/or metastasis to lymph nodes in multiple cancers, including colorectal [48] and breast [49-51]. VEGF-D is also involved in lymphangiogenesis and lymphatic metastasis [52,53].

#### Crosstalk between the VEGF signaling pathway and other angiogenic signaling pathways

The angiopoietins Ang-1, Ang-2, and Ang-4 bind to the receptor tyrosine kinases Tie1 and Tie2 on vascular endothelial cells and are involved in the angiogenic switch, metastasis, and lymphangiogenesis [6,31]. Ang-1 is expressed by mural cells, fibroblasts, and non-vascular normal and tumor cells, whereas Ang-2 is expressed primarily by endothelial cells and behaves in an autocrine manner [31]. Overexpression of Ang-2 has been shown to be associated with poor prognosis for a number of different cancers [31]. The Ang/Tie signaling pathway interacts with VEGF-A-mediated signaling in tumor angiogenesis [6,31]. Ang-2 is upregulated by proangiogenic factors, including VEGF-A, PDGFB, and insulin-like growth factor (IGF)1 [31] (Figure 2). Ang-2 causes pericytes to dissociate from endothelial cells in pre-existing vessels and, in the presence of VEGF-A, this leads to angiogenesis [6,31]. Ang-1 expressed by pericytes appears to help to maintain the integrity of blood vessels [31].

The Notch receptors Notch 1 and Notch 4 and the ligands jagged 1, Dll1, and Dll4 are expressed by vascular endothelial cells [27] and are involved in sprouting angiogenesis [31]. There are multiple modes of crosstalk between Notch/Dll4 and VEGF signaling (Figure 2). Dll4 expression and Notch signaling are induced by VEGF-A and VEGF-C [27,33]. VEGFR-3 regulates Notch signaling and the conversion of tip cells to stalk cells in sprouting angiogenesis [34]. Notch upregulates VEGFR-3 and allows VEGF-A/VEGFR-2-independent angiogenesis [35]. In addition, Dll4/Notch signaling may mediate resistance to anti-VEGF therapy through multiple different mechanisms, e.g. decreased levels of hypoxia-induced VEGF and increased levels of the VEGF receptor VEGFR1 in the tumor stroma, decreased levels of VEGFR2 in large blood vessels, and reduced levels of VEGFR3 overall [54].

Integrins and hypoxia may also have an impact on VEGF and other signaling components in tumor angiogenesis (Figure 2). αv integrins, which are expressed on multiple cell types, contribute to angiogenesis [32]. These and other integrins interact with the VEGF/VEGFR and Ang/Tie signaling pathways [32]. Hypoxia, through HIF-1α and HIF-2α, leads to increased expression of VEGF-A; hypoxia may also regulate PlGF expression, which may be more complicated. Basically, tissue hypoxia may not only elevate the abundance of VEGF-A, but may also increase other angiogenic regulatory factors, therefore leading to angiogenic activity alteration [26].

VEGF-A, often referred to as VEGF without a suffix [4], is the sole target of bevacizumab, a humanized monoclonal antibody approved for the treatment of colorectal and other cancers [55] and aflibercept (VEGF Trap, known in the US as ziv-aflibercept), is a recombinant fusion protein with receptor components of VEGFR-1 and VEGFR-2 that binds multiple ligands in the angiogenesis network (VEGF-A, VEGF-B, and PlGF) [56]. Aflibercept was recently approved for use by the US FDA with the US name of ziv-aflibercept, in combination with 5-fluorouracil, leucovorin, irinotecan (FOLFIRI) for patients with metastatic colorectal cancer (mCRC) that is resistant to or has progressed following an oxaliplatin-containing regimen. In addition to agents that target the various VEGFs and VEGFRs, agents that target other angiogenic mediators, including Ang-1 and −2, Notch signaling, HIF-1α, and integrins, and that are in clinical development are listed in the NCT registry (for example NCT01210222, NCT01193868, NCT01120288, and NCT00915278).

### Benefits of antiangiogenic agents in the treatment of metastatic colorectal cancer: preclinical and clinical evidence

As mentioned above, bevacizumab and aflibercept are antiangiogenic agents approved for the treatment of mCRC [55,56]. Other antiangiogenic therapies currently in late-stage clinical trials for mCRC include the anti-VEGFR-2 monoclonal antibody ramucirumab, and the tyrosine kinase inhibitor (TKI) regorafenib; additional TKIs (brivanib alaninate, cediranib, sunitinib, and vatalanib) had negative results in phase 3 trials (Table 1). Preclinical studies with these agents supported their further development for the treatment of colorectal cancer and other malignancies.

**Table 1:** Recent and ongoing phase 3 trials of antiangiogenic agents for the treatment of mCRC

**Trial**	**ClinicalTrials**.**gov identifier**
*Biologics*	
Bevacizumab in combination with crossover fluoropyrimidine-based chemotherapy beyond progression (stratum 1: AIO-IRI, FOLFIRI, CAPIRI or XELIRI; stratum 2: FUFOX, FOLFOX, CAPOX or XELOX) (ML 18147 study)*	NCT00700102
Aflibercept plus irinotecan and 5-FU following failure of an oxaliplatin-based regimen (VELOUR)	NCT00561470
Ramucirumab plus FOLFIRI following progression with first-line bevacizumab, oxaliplatin, and fluoropyrimidine	NCT01183780
*TKIs*	
Brivanib alaninate in combination with the EGFR monoclonal antibody cetuximab following previous chemotherapy	NCT00640471
Cediranib plus chemotherapy (FOLFOX or XELOX) as first-line treatment (HORIZON II)	NCT00399035
Cediranib plus FOLFOX vs bevacizumab plus FOLFOX as first-line treatment (HORIZON III)	NCT00384176
Regorafenib plus best supportive care following failure of standard therapies (CORRECT)	NCT01103323
Sunitinib plus FOLFIRI as first-line treatment	NCT00457691
Vatalanib plus oxaliplatin/5-FU/leucovorin following previous treatment with irinotecan	NCT00056446

*Note that there are additional phase 3 studies of bevacizumab for the treatment of mCRC.

TKIs = tyrosine kinase inhibitors.

#### Preclinical data

In preclinical models, bevacizumab demonstrated antitumor activity both as a single agent and in combination with chemotherapy or radiotherapy [57]. In tumor xenograft models, bevacizumab was shown to decrease tumor weight and size, tumor growth, vascular permeability, ascites formation, and the diameter and density of tumor vessels [57]. One of the proposed mechanisms by which bevacizumab is believed to enhance cytotoxic treatments is via “normalization” of the tumor vasculature [57], and the use of bevacizumab in combination with chemotherapy or radiotherapy has been shown to have at least additive activity in certain tumor models [57].

Aflibercept (VEGF Trap) is a recombinant fusion protein that contains domains from VEGFR-1 and VEGFR-2 and targets VEGF-A as well as VEGF-B and PlGF [58,59]. In certain tumor xenograft models, aflibercept inhibited the growth of new and established tumors and tumor angiogenesis; reduced vessel density, patency, and blood flow; and inhibited metastases and ascites formation [60]. Aflibercept also increased tumor hypoxia and decreased expression of tumor vascular genes and decreased activation of vascular endothelial signaling pathways [60]. In tumor xenografts, aflibercept in combination with other agents (radiotherapy, chemotherapy, or trastuzumab) showed greater inhibition of tumor growth and tumor vasculature than was observed with the individual agents alone [60].

In a recent study, the binding characteristics of bevacizumab and aflibercept were compared using several preclinical assessments [61]. Aflibercept showed tight binding to VEGF 165; dissociation constant (K_D_) was significantly lower with aflibercept compared with dimerized VEGFR1 or VEGFR2. Additionally, the K_D_ of aflibercept (0.490 pM) was approximately 100-fold lower compared with that of bevacizumab (58 pM), suggesting a 100-fold tighter binding to VEGF 165 [61].

Differences in biologic activity were also demonstrated preclinically. In a study of VEGF-A–induced activation of VEGFR1, aflibercept demonstrated 92-fold greater potency than bevacizumab in an assay in which VEGFR1 activation was induced by VEGF-A 165 or VEGF-A 121 [61]. Aflibercept also inhibited VEGFR1 activation by PlGF2, [61]. Aflibercept also inhibited activation of VEGFR2 activation induced by VEGF-A 165 or VEGF-A 121, which may suggest some clinical benefits since binding kinetics and affinity are important determinants of the biological activity of antibody-like drugs [61].

Ramucirumab, a fully human monoclonal antibody against VEGFR-2, was designed to bind to a VEGFR-2 epitope involved in ligand binding [62]. It has shown anticancer activity alone and in combination with other agents in preclinical models of leukemia, solid tumors, and metastases [63-65].

Various antiangiogenic TKIs have also demonstrated preclinical activity in cancer models. Cediranib, a VEGFR-2 TKI, inhibited tumor growth and reduced microvessel density in tumor xenograft models [66]. Regorafenib, a multikinase inhibitor whose targets include VEGFR-1-3 and Tie2, induced tumor growth inhibition or shrinkage and reduced extravasation in tumor xenograft models [67,68]. Brivanib, a dual VEGFR-2/FGFR-1 kinase inhibitor, reduced tumor proliferation, vascular density, and microcirculation; inhibited VEGF- and FGF-driven angiogenesis; and induced apoptosis in tumor xenograft models [69,70]. Sunitinib, a multikinase inhibitor that targets VEGFR-2, PDGFR, KIT, and FLT3, inhibited VEGFR-2 phosphorylation and VEGF-induced vascular permeability and caused tumor regression, growth arrest, or growth inhibition in tumor xenografts [71]. Vatalanib, an inhibitor of VEGFRs and other kinases, decreased tumor growth, metastasis, microvascular density, and blood flow in tumor xenograft models and induced tumor cell apoptosis [72-74].

Based on these preclinical data demonstrating anticancer activity, these agents moved forward into clinical studies for mCRC and other cancers.

#### Clinical data

##### Bevacizumab

The clinical benefit of antiantiogenesis agents in treatment of mCRC has been established. Based on encouraging data of a 3-arm phase 2 study [75], several pivotal phase 3 trials demonstrated that bevacizumab improved overall survival as first- or second-line therapy in combination with fluoropyrimidine-containing regimens (either combined with oxaliplatin or irinotecan) in patients with mCRC [76,77]. In the initial phase 3 pivotal trial, median overall survival (mOS) was increased from 15.6 months in patients who received IFL (bolus 5-FU, leucovorin, and irinotecan), but only to 20.3 months in patients who received IFL + bevacizumab (*P*<0.001) as first line therapy in mCRC [76]. The E3200 study showed the benefit of bevacizumab as second line therapy when combined with FOLFOX (infusional 5-FU and oxaliplatin) with mOS of 10.8 vs. 12.9 months (*P*=0.0018) [77]. Together, these trials confirmed a survival benefit with bevacizumab in both the first- and second-line settings for mCRC. Bevacizumab-associated toxicities identified in early trials of bevacizumab included hemorrhage, thromboembolism, proteinuria, and hypertension [76]. In phase 3 trials of bevacizumab plus chemotherapy in patients with mCRC, the incidence of grade ≥3 bleeding/hemorrhage was 2% to 3.4% with bevacizumab versus <1% to 2.5% with comparator; the incidence of grade ≥3 thromboembolism (reported in 2 trials) was 3.4% to 10% with bevacizumab versus 2.5% to 6% with comparator; the incidence of grade ≥3 venous thromboembolic events (VTEs, reported in 1 trial) was 8% with bevacizumab versus 5% with comparator; the incidence of grade ≥3 proteinuria was <1% in either arm; and the incidence of grade ≥3 hypertension was 4% to 11% with bevacizumab versus 1% to 2.3% with comparator [76-78]. Gastrointestinal perforation was also reported in phase 3 trials of bevacizumab in patients with mCRC: <1% to 1.5% with bevacizumab versus 0% to <1% with comparator [76-78].

Since antiangiogenic agents are not traditional cytotoxic chemotherapy agents, the question remains whether their antitumor efficacy may be maintained after patients’ disease has progressed after a bevacizumab-containing chemotherapy regimen. A registry study which suggested a significant survival benefit was controversial because of the registry study design [79]. There were no randomized study data until recently. In the TML (ML 18147) study, all patients were previously treated with bevacizumab in combination with either FOLFOX or FOLFIRI. After disease progression, they were then randomized to chemotherapy regimens they were not exposed to (either FOLFIRI or FOLFOX) with continuous bevacizumab. The overall survival was significantly improved with continuous bevacizumab plus chemotherapy versus chemotherapy alone as second line in mCRC patients who had progressed on first-line bevacizumab-containing regimens (11.2 versus 9.8 months, *P*=0.0211) [80]. This study validates the importance of continued antiangiogenic therapy in mCRC patients following progression.

##### Regorafenib

Another randomized phase 3 study (CORRECT trial) with a total 760 patients who were treated with standard therapies including bevacizumab-containing regimens demonstrated the efficacy of regorafenib in both overall survival (6.4 versus 5.0 months, HR=0.773, *P*=0.0051) and progression-free survival (1.9 versus 1.7 months, HR=0.493, *P*<0.000001) compared with placebo; the most common grade ≥3 adverse events were hand-foot reaction (17%), fatigue (15%), diarrhea (8%), hyperbilirubinemia (8%), and hypertension (7%) [81]. This study was the first to demonstrate the efficacy of an oral TKI in prolonging survival in patients with mCRC.

##### Aflibercept

Data from a phase 3 trial (VELOUR) (N=1226, with 28.3% of patients having had previous bevacizumab exposure) demonstrated that aflibercept plus FOLFIRI in patients with mCRC who had progressed following an oxaliplatin containing regimen significantly improved overall survival (13.5 months versus 12.06 months, HR=0.817, *P*=0.0032) and progression-free survival (6.90 months versus 4.67 months, HR=0.758, *P*=0.00007) compared with placebo plus FOLFIRI in mCRC patients previously treated with FOLFOX [82,83]. These data led to the FDA approval of aflibercept (ziv-aflibercept) for the treatment of mCRC following oxaliplatin-based chemotherapy. While the progression-free survival benefit remained in the prior bevacizumab-treated patients (6.7 versus 3.9 months, HR=0.661 [95% CI 0.512-0.852]) based on the prespecified subgroup analysis, the study was not powered to show a treatment difference between arms; therefore, no definitive conclusions may be drawn concerning the benefit of aflibercept in the prior bevacizumab-treated subgroup. The most common grade 3–4 adverse events with more than 2% higher incidence with aflibercept were diarrhea, asthenia/fatigue, stomatitis/ulceration, infections, hypertension, gastrointestinal/abdominal pains, neutropenia/neutropenic complications, and proteinuria.

##### Cediranib

In the HORIZON II trial, cediranib plus chemotherapy (FOLFOX or XELOX) significantly improved progression-free survival (HR=0.84) but not overall survival (HR=0.94) compared with chemotherapy plus placebo; treating to progression with cediranib plus chemotherapy appeared to have a beneficial effect [84].

##### Brivanib

In the NCIC Clinical Trials Group and AGITG CO.20 trial, brivanib alaninate plus cetuximab significantly improved progression-free survival (5.0 versus 3.4 months, HR=0.72, *P*<0.0001) but not overall survival (8.8 versus 8.1 months, HR=0.88, *P*=0.12) compared with cetuximab plus placebo; the most frequent grade ≥3 adverse events with brivanib alaninate were fatigue (25%), hypertension (11%), and rash (5%) [85].

## Conclusions

The importance of angiogenesis in cancer, and an increased understanding of the network of signaling pathways that mediate it, have led to the development of a number of antiangiogenic agents for the treatment of cancer. Bevacizumab is approved for the first- or second-line treatment of mCRC when added to intravenous 5-fluorouracil-based regimens, and aflibercept was approved by the FDA when added to FOLFIRI in patients with mCRC previously treated with an oxaliplatin-based regimen. Other antiangiogenic agents are in late-stage clinical development. The addition of bevacizumab or aflibercept to chemotherapy in patients with mCRC has demonstrated improved overall survival compared with chemotherapy alone, and regorafenib added to best supportive care has demonstrated improved survival compared with placebo. Insight into how angiogenic signaling pathways intersect may aid in the design of agents with improved efficacy and safety profiles and a reduced risk of resistance. Additional research is needed regarding how to sequence and combine approved and investigational antiangiogenic agents for the treatment of colorectal and other cancers.

## Competing interest

I have participated in the advisors meetings of Genentech and Sanofi in the past 1-2 years.
